# A Clinical Perspective of Anti-Fibrotic Therapies for Cardiovascular Disease

**DOI:** 10.3389/fphar.2017.00186

**Published:** 2017-04-06

**Authors:** Lu Fang, Andrew J. Murphy, Anthony M. Dart

**Affiliations:** ^1^Baker IDI Heart and Diabetes InstituteMelbourne, VIC, Australia; ^2^Department of Cardiovascular Medicine, The Alfred HospitalMelbourne, VIC, Australia

**Keywords:** cardiac fibrosis, anti-fibrotic therapies, clinical trials, diffuse fibrosis, cardiac magnetic resonance imaging, collagen turnover markers, diastolic function

## Abstract

Cardiac fibrosis are central to various cardiovascular diseases. Research on the mechanisms and therapeutic targets for cardiac fibrosis has advanced greatly in recent years. However, while many anti-fibrotic treatments have been studied in animal models and seem promising, translation of experimental findings into human patients has been rather limited. Thus, several potential new treatments which have shown to reduce cardiac fibrosis in animal models have either not been tested in humans or proved to be disappointing in clinical trials. A majority of clinical studies are of small size or have not been maintained for long enough periods. In addition, although some conventional therapies, such as renin-angiotensin-aldosterone system (RAAS) inhibitors, have been shown to reduce cardiac fibrosis in humans, cardiac fibrosis persists in patients with heart failure even when treated with these conventional therapies, indicating a need to develop novel and effective anti-fibrotic therapies in cardiovascular disease. In this review article, we summarize anti-fibrotic therapies for cardiovascular disease in humans, discuss the limitations of currently used therapies, along with possible reasons for the failure of so many anti-fibrotic drugs at the clinical level. We will then explore the future directions of anti-fibrotic therapies on cardiovascular disease, and this will include emerging anti-fibrotics that show promise, such as relaxin. A better understanding of the differences between animal models and human pathology, and improved insight into carefully designed trials on appropriate end-points and appropriate dosing need to be considered to identify more effective anti-fibrotics for treating cardiovascular fibrosis in human patients.

## Introduction

Cardiac fibrosis is a hallmark of various cardiovascular disease such as hypertension, myocardial infarction (MI), and ischemic, dilated, and hypertrophic cardiomyopathies. Cardiac fibrosis not only leads to cardiac diastolic dysfunction, but is also a major determinant of malignant arrhythmias and end-stage systolic heart failure and consequently, increases the risk of cardiac death. There are two types of fibrosis: regional fibrosis (reparative fibrosis, scarring from MI) and diffuse fibrosis (reactive fibrosis, interstitial fibrosis in response to different stimuli). Cardiac fibrosis can be definitively diagnosed by endomyocardial biopsies, but they are an invasive evaluation only representative in diffuse fibrosis. Circulating biomarkers, particularly collagen turnover markers, are widely used to noninvasively assess cardiac fibrosis, however they are not reliable and unable to differentiate regional fibrosis from diffuse fibrosis. Cardiac magnetic resonance imaging (CMR) is an emerging technique to accurately and noninvasively evaluate regional and diffuse cardiac fibrosis by late gadolinium enhancement and post-contrast myocardial longitudinal relaxation time (T1) mapping, respectively, but CMR is expensive and not easily accessible. Functional consequences of cardiac fibrosis, particularly impaired left ventricular (LV) relaxation and heart failure, are also potential, albeit nonspecific, markers of fibrosis.

It is known that a complex interaction involving a network of growth factors/cytokines/hormones and fibroblasts and other cell types (such as cardiomyocytes, monocytes, lymphocytes) is responsible for initiating and maintaining fibrotic response (Kong et al., 2014). In addition to resident fibroblasts, fibroblasts originate from circulating pecursors, sometimes termed fibrocytes (Fang et al., 2013), endothelial cells or epithelial cells (Kong et al., 2014). Renin-angiotensin-aldosterone system (RAAS), growth factors [such as transforming growth factor (TGF)-β], endothelin, matricellular proteins (such as connective tissue growth factor (CTGF) and proinflammatory factors (such as tumor necrosis factor (TNF-α), interleukin (IL)-6 and IL-1) are some of the best studied mediators implicated in cardiac fibrosis (Kong et al., 2014). Recently, new mediators with therapeutic potential of cardiac fibrosis have been emerging such as cardiotrophin-1, galectin, and miRNAs etc. (Fang et al., 2015; Heymans et al., 2015). Although many anti-fibrotic therapies on cardiac fibrosis seem promising in experimental models, clinical data are limited and mixed. Most of new anti-fibrotic therapies have not been evaluated in patients. Some clinical data have demonstrated benefits on cardiac fibrosis mainly with RAAS inhibitors, but most clinical trials on anti-fibrotic drugs are disappointing. This review will summarize findings from clinical trials of anti-fibrotic therapies on cardiac fibrosis (Table 1) and discuss the discrepancy between animal research and clinical trials as well as future directions.

**Table 1:** **Anti-fibrotic therapies on cardiac fibrosis in clinical trials**.

**Study**	**Agent**	**Length of treatment**	**Patient included (*n*)**	**Main findings**
**RAAS INHIBITORS**				
Brilla et al., 2000	Lisinopril	6 months	35	Lisinopril but not hydrochlorothiazide decreased CVF in hypertensive patients.
López et al., 2001	Losartan	12 months	37	Losartan but not amlodipine decreased CVF and PICP in hypertensive patients.
Díez et al., 2002	Losartan	12 months	19	Losartan decreased CVF and LV chamber stiffness in hypertensive patients with severe fibrosis, but not in those with nonsevere fibrosis.
Shibasaki et al., 2005	Losartan	6 months	39	Losartan more effectively suppressed cardiac fibrosis than enalapril or amlodipine in patients with end-stage renal disease.
Shimada et al., 2013	Losartan	12 months	20	Losartan attenuated the progression of cardiac fibrosis in patients with nonobstructive hypertrophic cardiomyopathy.
Kawamura et al., 2010	Candesartan	24 months	153	Candesartan reduced PIIINP in patients with atrial fibrillation.
Kosmala et al., 2011	Spironolactone	6 months	80	Additional spironolactone decreased PICP and PIIINP in patients with metabolic syndrome.
Kosmala et al., 2013	Spironolactone	6 months	113	Spironolactone improved myocardial deformation and decreased PICP and PIIINP in patients with obesity and mild LV diastolic dysfunction.
Mak et al., 2009	Eplerenone	12 months	44	Eplerenone reduced PIIINP and modestly improved diastolic function in patients with diastolic heart failure.
Deswal et al., 2011	Eplerenone	6 months	44	Eplerenone reduced PINP and PICP in patients with heart failure with preserved ejection fraction.
**INFLAMMATION MODULATORS**				
RENEWAL	Etanercept	24 weeks	2,048	The study ruled out a clinically relevant benefit of etanercept on the rate of death or hospitalization due to chronic heart failure in patients with heart failure.
ATTACH	Infliximab	At 0, 2, 6 weeks	150	High dose of infliximab increased all-cause mortality in patients with moderate-severe heart failure.
Abulhul et al., 2012	Atorvastatin	6 months	56	Atorvastatin reduced PIIINP in heart failure patients.
CORONA	Rosuvastatin	32.8 months	5,011	Rosuvastatin did not reduce the primary outcome or the number of deaths from any cause in older patients with systolic heart failure.
GISSIF-HF	Rosuvastatin	3.9 years	4,574	Rosuvastatin daily did not affect clinical outcomes in patients with chronic heart failure of any cause.
UNIVERSAL	Rosuvastatin	6 months	86	Rosuvastatin did not beneficially alter parameters of LV remodeling in patients with chronic systolic heart failure.
**TGF-β INHIBITORS**				
PRESTO	Tranilast	1, or 3 months	11,484	Tranilast did not improve the quantitative measures of restenosis (angiographic and intravascular ultrasound) or its clinical sequelae in patients receiving successful percutaneous coronary intervention.
**ENDOTHELIN INHIBITORS**				
Sütsch et al., 1998	Bosentan	2 weeks	36	Bosentan improved systemic and pulmonary hemodynamics in heart failure patients who were symptomatic with standard triple-drug therapy.
EARTH	Darusentan	24 weeks	642	Darusentan did not improve cardiac remodeling or clinical outcomes in patients with chronic heart failure.
Prasad et al., 2006	Enrasentan	6 months	72	In asymptomatic patients with LV dysfunction, LVEDVI increased over 6 months with enrasentan compared with enalapril treatment.
**SELECTIVE HEART RATE-REDUCING DRUG**				
SHIFT	Ivabradine	22.9 month follow up	6,558	Ivabradine improved clinical outcomes in patients with symptomatic heart failure.
SHIFT substudy	Ivabradine	8 month follow up	411	Ivabradine reversed cardiac remodeling in patients with heart failure.
**LOOP DIURETICS**				
López et al., 2004	Torsemide	8 months	36	Torsemide but not furosemide reduced PICP and CVF in hypertensive patients with symptomatic heart failure.
López et al., 2007	Torsemide	8 months	22	Torsemide but not furosemide decreased PCP in patients with chronic heart failure.
López et al., 2009	Torsemide	8 months	24	Torsemide corrected both lysyl oxidase overexpression and enhanced collagen cross-linking leading to normalization of LV chamber stiffness in patients with heart failure.
TORAFIC	Torsemide	8 months	155	In hypertensive patients with chronic heart failure randomized to torsemide or furosemide, there were no difference in PICP levels between the two groups.
**CYCLIC GMP-SPECIFIC PHOSPHODIESTERASE TYPE-5A INHIBITOR**				
Giannetta et al., 2012	Sildenafil	3 months	59	Sildenafil improved LV contraction parameters and reduced TGF-β and MCP-1 in patients with diabetic cardiomyopathy.
Redfield et al., 2013	Sildenafil	24 weeks	216	Sildenafil did not improve exercise activity in patients with heart failure with preserved ejection fraction.
**MATRIX METALLOPROTEINASE INHIBITOR**				
PREMIER	PG-116800	90 days	253	PG-11680 did not prevent LV remodeling or improve clinical outcomes 90 days after myocardial infarction.
**RELAXIN**				
Pre-RELAX-AHF	Relaxin	48 h	234	Relaxin improved dyspnoea and lowered cardiovascular deaths or readmissions due to heart or renal failure at day 60 in patients with acute heart failure.
RELAX-AHF	Serelaxin	48 h	1,161	Serelaxin improved dyspnoea and reduced cardiovascular deaths and all-cause mortality through day 180 in patients with acute heart failure.

*CVF, collagen volume fraction; PICP, the carboxy-terminal peptide of procollagen type I; LV, left ventricular; PIIINP, the amino-terminal peptide of type III procollagen; PINP, the amino-terminal peptide of type I procollagen (PINP); PCP, procollagen type I carboxy-terminal proteinase; TGF-β, transforming growth factor-β; MCP-1, monocyte chemoattractant protein-1; LVEDVI, LV end diastolic volume index*.

## Anti-fibrotic therapies on cardiac fibrosis in clinical trials

### RAAS inhibitors

The first family of anti-fibrotic drugs are inhibitors of angiotensin II. Angiotensin II interacts with angiotensin II type I receptors, which stimulates fibroblast proliferation, and increases collagen synthesis (Kong et al., 2014). Several clinical studies have shown that both angiotensin-converting enzyme (ACE) inhibitors and angiotensin receptor blockers reduce cardiac fibrosis in patients independent of their antihypertensive effects. In hypertensive patients, endomyocardial biopsies at baseline and 6 months revealed a decrease of collagen volume fraction (CVF) only in the group treated with lisinopril (*n* = 18), but not with hydrochlorothiazide (*n* = 17) (Brilla et al., 2000). A comparison between losartan (*n* = 21) and amlodipine (*n* = 16) given for 1 year in hypertensive patients revealed that only losartan significant decreased both CVF (by endomyocardial biopsies) and the carboxy-terminal peptide of procollagen type I (PICP) (López et al., 2001). Another study demonstrated that in patients with hypertensive heart disease, losartan treatment for 12 months decreased CVF (by endomyocardial biopsies) and LV chamber stiffness in patients with severe fibrosis (*n* = 7), but not in those with nonsevere fibrosis (*n* = 12) (Díez et al., 2002). In patients with end-stage renal disease, losartan (*n* = 13) more effectively suppressed cardiac fibrosis than did enalapril (*n* = 13) or amlodipine (*n* = 13) (Shibasaki et al., 2005). Another small study showed attenuation of progression of cardiac fibrosis with losartan in patients with nonobstructive hypertrophic cardiomyopathy (Shimada et al., 2013). Treatment with candesartan for 24 months also reduced the amino-terminal peptide of type III procollagen (PIIINP) in patients with atrial fibrillation (Kawamura et al., 2010).

The mineralo-corticoid receptor antagonists, spironolactone and eplerenone, also have anti-fibrotic effects in humans. Additional treatment of spironolactone for 6 months improved LV diastolic function and decreased PICP and PIIINP in 80 patients with metabolic syndrome treated with angiotensin II inhibition (Kosmala et al., 2011). In another study of 113 patients with obesity and mild LV diastolic dysfunction, spironolactone treatment for 6 months improved myocardial deformation and decreased PICP and PIIINP (Kosmala et al., 2013). In 44 patients with diastolic heart failure, eplerenone reduced PIIINP at 12 months after treatment, associated with modest improvement of diastolic function (Mak et al., 2009). Similar findings were made in another study showing that eplerenone reduced the amino-terminal peptide of type I procollagen (PINP) and PICP in 44 patients with heart failure with preserved ejection fraction (Deswal et al., 2011).

Although the above clinical studies have shown that RAAS inhibitors reduces cardiac fibrosis in humans, the study population in these studies is rather small. Furthermore, inhibition of RAAS only modestly regresses cardiac fibrosis. Cardiac fibrosis persists in heart failure patients even when treated as recommended by the official guidelines (Querejeta et al., 2004). Thus, there is a compelling need to develop novel and effective anti-fibrotic therapies in cardiovascular disease.

### Inflammation modulators

Inflammatory modulation might have beneficial effects on cardiac fibrosis and heart failure since inflammation is involved in the formation and progression of cardiac fibrosis. TNF-α plays an important role in cardiac fibrosis. However, the RENEWAL study (Mann et al., 2004) which examined the effect of TNF-α antagonist etanercept in patients with heart failure was negative. Additionally, the ATTACH trial was stopped prematurely as the high dose of the TNF-α antagonist infliximab increased all-cause mortality in patients with moderate-to-severe chronic heart failure (Chung et al., 2003). The finding that TNF receptor 1 and 2 exert opposing effects on cardiac remodeling may partly explain that direct blockade of one inflammatory actor could cause these unexpected clinical results (Hamid et al., 2009).

Statins possess potent anti-inflammatory effects and are widely used in cardiovascular disease. Rosuvastatin attenuated cardiac fibrosis in animal models (Chang et al., 2009), which is supported by a small clinical study showing that statin therapy for 6 months reduced PIIINP in heart failure population (*n* = 56) (Abulhul et al., 2012). However, two large-scale clinical trials, the CORONA(Kjekshus et al., 2007) and GISSIF-HF (Tavazzi et al., 2008) observed a neutral effect of rosuvastatin compared to placebo on major clinical outcomes in heart failure. Beneficial effects of statin on cardiac remodeling were not observed in universal trial either (Krum et al., 2007). A sub-study of universal trial actually showed reduced coenzyme-10 and increased serum collagen markers in the statin-treated group (Ashton et al., 2011). So, statin's effect on cardiac fibrosis in human are generally disappointing. Peroxisome proliferator-activated receptor (PPAR) agonists have anti-inflammation properties. Preclinical data showed that PPAR-α agonist inhibited cardiac fibrosis and improved cardiac function (Ogata et al., 2004). However, considerable controversy exists on the cardiac safety profile of PPAR agonists (Sarma, 2012). Overall, there is lack of effective inflammatory modulators to inhibit cardiac fibrosis in patients. However, the negative results of inflammatory modulators do not necessarily mean the end of inflammatory modulators in cardiac fibrosis. Future studies should identify the crucial actors and their mechanisms of action in the immunopathogenesis of cardiac fibrosis, which is a prerequisite for the development of new inflammatory modulators in patients with cardiac fibrosis. Selective p38 MAPK inhibitors blocking the secretion of TNF-α and decreasing cardiac fibrosis in mice (Westermann et al., 2006) may be a new treatment modality in humans.

### TGF-β inhibitors

TGF-β plays a central role in activating cardiac fibrosis and it activates both canonical (ALK/Smad2/3/Smad4) and noncanonical (TAK/p-38/JNK and NOX4/ROS) signaling pathways. Anti-TGF-β antibodies and ALK5 inhibitors attenuated cardiac fibrosis in animal models, but they were associated with adverse cardiovascular effects (Frantz et al., 2008; Engebretsen et al., 2014), suggesting that targeting canonical TGF-β signaling pathway might not be applicable clinically. While TGF-β promotes fibrogenesis, it also inhibits inflammation, suggesting that broad targeting of TGF-β may be problematic. Alternatively, targeting TAK or NOX4 downstream of TGF-β might be viable anti-fibrotic approaches. Clinically, two agents, pirfenidone and tranilast, which inhibit TGF-β and other growth factors (Edgley et al., 2012), have been available. Both pirfenidone and tranilast have been shown to reduce cardiac fibrosis in animal studies (Edgley et al., 2012). However, tranilast was disappointing in the PRESTO study for post-percutaneous transluminal coronary angioplasty restenosis prevention (Holmes et al., 2002). The use of pirfenidone and tranilast also have adverse effects such as liver dysfunction. Now research is being conducted to search for new compounds that could overcome these potential safety concerns. A new compound called FT011 displays improved activity and reduced toxicity compared to tranilast (Zammit et al., 2009), which needs to be investigated in clinical studies.

### Endothelin inhibitors

Endothelin is another important contributor of fibrotic process and bosentan, a dual endothelin receptor subtype A and B antagonist, prevents fibrosis of various organs in animal models (Clozel and Salloukh, 2005). Dual endothelin subtype A and B inhibitors bosentan and macitentan and the ETA inhibitor ambrisentan are approved in the U.S. for the treatment of pulmonary hypertension. An initial small study in human showed that additional administration of bosentan improved systemic and pulmonary hemodynamics in severe heart failure patients receiving conventional treatments including ACE inhibitors (Sütsch et al., 1998). However, most of subsequent clinical trials of endothelin receptor antagonists were negative or neutral (Anand et al., 2004; Prasad et al., 2006). The harmful effects of endothelin receptor antagonists were generally attributable to enhanced fluid retention, which could be alleviated by early diuretic therapy. However, in general, additional blockade of endothelin may not be beneficial in patients with heart failure or cardiac fibrosis receiving angiotensin inhibitors.

### β-blockers

β-blockers have been demonstrated to prevent cardiac fibrosis and improve survival in a rat model (Kobayashi et al., 2004). A meta-analysis showed that the β-blockers treatment for the patients with heart failure with preserved ejection fraction was associated with a lower risk of all-cause mortality (Liu et al., 2014). However, the mechanisms of β-blockers' benefit on mortality have not been precisely clarified and whether β-blockers attenuate cardiac fibrosis in human remains unknown.

### Selective heart rate-reducing treatment: ivabradine

Ivabradine is an oral medication that provides selective heart rate reduction by inhibiting the f-channel. A large trial SHIFT showed that over a median follow-up of 22.9 months, ivabradine significantly reduced cardiovascular death or hospital admission for worsening heart failure in patients with symptomatic heart failure with an LV ejection fraction ≤35%, and in sinus rhythm with a heart rate of ≥70 bpm (Swedberg et al., 2010). An echocardiographic sub-study of SHIFT further found that ivabradine improved both LV end-systolic and end-diastolic volume indexes compared with placebo from baseline to the 8-month follow-up (Tardif et al., 2011). Thus, ivabradine has been introduced in the treatment guidelines for chronic heart failure in patients (McMurray et al., 2012). However, evidence on whether ivabradine attenuates cardiac fibrosis in patients with heart failure is still lacking although ivabradine effectively reduced fibrosis and circulating angiotensin II and aldosterone levels in animal models (Busseuil et al., 2010). Ivabradine could also reduce fibrosis through its inhibitory effects on inflammatory responses and cardiac apoptosis (Becher et al., 2012).

### Loop diuretics: torsemide

There are three loop diuretics utilized in heart failure patients: furosemide, torsemide, and bumetanide. López et al. reported that torasemide (*n* = 19), but not furosemide (*n* = 17), reduced circulating PICP and myocardial collagen in hypertensive patients with symptomatic heart failure (López et al., 2004). They then found that activation of the enzyme responsible for the cleavage of PICP, procollagen type I carboxy-terminal proteinase (PCP), was also decreased in 22 patients with chronic heart failure taking torasemide (López et al., 2007). They further reported the ability of torsemide to correct both lysyl oxidase overexpression and enhanced collagen cross-linking leading to normalization of LV chamber stiffness in patients with heart failure (López et al., 2009). This was supported by preclinical data showing torsemide's effect on RAAS inhibition including decreasing aldosterone secretion, inhibiting aldosterone receptor and Ang II effects (Buggey et al., 2015). However, in the TORAFIC study, a multi-center study of 155 hypertensive patients with chronic heart failure randomized to torsemide or furosemide, investigators did not find significant differences between the two groups in changes of PICP (Group, 2011). The TORAFIC study's patient population had less severe heart failure and lower baseline serum PICP compared to those in the studies by Lopez and colleagues, which possibly explains the divergent results among these studies. So, it is important to select patients who may benefit from torsemide treatment.

### Sildenafil

Sildenafil inhibits cyclic GMP-specific phosphodiesterase type-5A and it has been used to treat idiopathic pulmonary fibrosis. In the first proof-of-concept human study, 59 patients with isolated diabetic cardiomyopathy randomly treated 3 months with sildenafil showed improved LV contraction parameters and reduced TGF-β and monocyte chemoattractant protein-1, when compared with controls (Giannetta et al., 2012). However, among patients with heart failure with preserved ejection fraction sildenafil for 24 weeks (*n* = 113), compared with placebo (*n* = 103), did not improve exercise capacity or clinical status (Redfield et al., 2013). Notably, fibrosis parameters were not measured in these two studies. The discordant results indicate that the same treatment does not exert similar effects in various cardiovascular disease.

### Matrix metalloproteinase (MMP) inhibitors

Cardiac fibrosis is associated with activation of MMPs. It has been shown that MMP inhibition attenuates cardiac fibrosis and LV remodeling in experimental models (Heymans et al., 2005; Matsusaka et al., 2006). However, the PREMIER study of an orally active MMP inhibitor, PG-116800, in 253 patients after M failed to prevent LV remodeling or improve clinical outcomes 90 days after MI (Hudson et al., 2006), although it should be noted that no fibrosis parameters were measured in this study.

### Relaxin

Relaxin is an intriguing endogenous hormone that is a potent vasodilator with a number of pleiotropic effects. Relaxin inhibits fibrosis through various mechanisms including inhibiting TGF-β and Smad, regulating the balance between MMPs and tissue inhibitors of metalloproteinases, and inhibiting inflammatory response (Samuel et al., 2016). Relaxin has been shown to have anti-fibrotic effects in a range of experimental models of cardiovascular disease including MI (Samuel et al., 2011), fibrotic cardiomyopathy (Samuel et al., 2014), hypertension (Lekgabe et al., 2005), diabetes (Samuel et al., 2008), and atrial fibrillation (Henry et al., 2016). Furthermore, relaxin more effectively ameliorated cardiac fibrosis than enalapril in an experimental model of fibrotic cardiomyopathy and relaxin in combination with enalapril augmented the anti-fibrotic efficacy of enalapril (Samuel et al., 2014). However, relaxin is not universally beneficial in cardiac fibrosis since relaxin did not affect pressure overload-induced cardiac fibrosis that was associated with biochemical wall stress rather than elevated TGF-β1 levels (Xu et al., 2008). The beneficial effects of a single 48-h infusion of relaxin in the acute heart failure trials (Teerlink et al., 2009, 2013) has led to great interest in its clinical application in in human disease. Although anti-fibrotic effects of relaxin is well characterized in various experimental models, clinical trials have failed in patients with other fibrotic conditions such as scleroderma (Khanna et al., 2009). The negative results of clinical trials of relaxin have pointed to the challenges previously underscored. First, relaxin has short *in vivo* half-life and it is costly to produce. The relatively short duration of relaxin treatment have partly contributed to the failed clinical trials since fibrosis is a slow process in human. Second, it is important to know the expression of relaxin receptors in different tissues and organs and to understand tissue competence to respond to relaxin along with signaling pathways. Nevertheless, relaxin still holds great potential as a therapy for cardiac fibrosis associated with various cardiovascular disease.

## Challenges and future directions

The failure of many clinical trials on anti-fibrotic drugs indicates that extrapolating research data from animal models to human requires caution since there are significant species differences in physiology and genetics between animals and human. Compared to mice, fibrosis is a slower condition in humans, which takes decades to develop and require long-term treatment to diminish its progression. Furthermore, many animal models of diseases do not mimic various clinical settings, thus controversial results are likely to be obtained since there are different signaling pathways and mechanisms in cardiac fibrotic processes in various diseases. In addition, the animals used are normally young while patients with cardiac fibrosis are at a more advanced age. In order to improve clinical translation, it is important to design, conduct and analyse animal experiments properly and to summarize data from animal research adequately before conducting clinical trials. Moreover, the failure of previous trials also emphasizes the need for optimal design of future clinical trials including a selection of suitable patients, appropriate dose, and route and timing and length of administration.

There are some important areas for future research in this field. First, although CMR is too expensive to be used in large populations, it should be used to investigate potential new treatments with relatively small group sizes since it allows accurate assessment of regional and diffuse fibrosis. Second, anti-fibrotic therapies targeting downstream signaling pathways may improve safety and efficacy of current treatments such as TGF-β inhibitors. In addition to their role in fibrosis, many proteins are involved in other biological processes. Thus, developing more specific agents targeting fibrotic signaling pathways is likely to be beneficial to minimize potential side effects. Third, combined anti-fibrotic therapies seem more effective than single drug treatment. It is shown that spironolactone or relaxin in combination with angiotensin II inhibitors augmented anti-fibrotic efficacy (Kosmala et al., 2011; Samuel et al., 2014). Combined anti-fibrotic agents with different mechanisms of actions is likely to exert better effects on cardiac fibrosis.

## Conclusion

Although many fibrotic therapies on cardiac fibrosis are promising in preclinical models, clinical translation is limited. There is still a lack of effective treatments to regress cardiac fibrosis in patients with various cardiovascular disease. Future optimally designed clinical studies are required to test new potential treatments and currently available drugs with improved safety and efficacy after adequate analysis of evidence from animal research.

## Author contributions

LF was responsible for assembling and drafting of the manuscript. AM and AD contributed to the drafting of the manuscript.

### Conflict of interest statement

The authors declare that the research was conducted in the absence of any commercial or financial relationships that could be construed as a potential conflict of interest.
